# Personas for Better Targeted eHealth Technologies: User-Centered Design Approach

**DOI:** 10.2196/24172

**Published:** 2022-03-15

**Authors:** Iris ten Klooster, Jobke Wentzel, Floor Sieverink, Gerard Linssen, Robin Wesselink, Lisette van Gemert-Pijnen

**Affiliations:** 1 Faculty of Behavioural, Management and Social Sciences University of Twente Enschede Netherlands; 2 Department of Health and Social Studies Windesheim University of Applied Sciences Zwolle Netherlands; 3 Research Group IT Innovations in Health Care Windesheim University of Applied Sciences Zwolle Netherlands; 4 Carintreggeland Hengelo Netherlands; 5 Hospital Group Twente Almelo and Hengelo Netherlands; 6 Location AMC Amsterdam University Medical Centers Amsterdam Netherlands

**Keywords:** personas, clustering, heart failure, eHealth, user-centered design

## Abstract

**Background:**

The full potential of eHealth technologies to support self-management and disease management for patients with chronic diseases is not being reached. A possible explanation for these lacking results is that during the development process, insufficient attention is paid to the needs, wishes, and context of the prospective end users. To overcome such issues, the user-centered design practice of creating personas is widely accepted to ensure the fit between a technology and the target group or end users throughout all phases of development.

**Objective:**

In this study, we integrate several approaches to persona development into the Persona Approach Twente to attain a more holistic and structured approach that aligns with the iterative process of eHealth development.

**Methods:**

In 3 steps, a secondary analysis was carried out on different parts of the data set using the Partitioning Around Medoids clustering method. First, we used health-related electronic patient record data only. Second, we added person-related data that were gathered through interviews and questionnaires. Third, we added log data.

**Results:**

In the first step, 2 clusters were found, with average silhouette widths of 0.12 and 0.27. In the second step, again 2 clusters were found, with average silhouette widths of 0.08 and 0.12. In the third step, 3 clusters were identified, with average silhouette widths of 0.09, 0.12, and 0.04.

**Conclusions:**

The Persona Approach Twente is applicable for mixed types of data and allows alignment of this user-centered design method to the iterative approach of eHealth development. A variety of characteristics can be used that stretches beyond (standardized) medical and demographic measurements. Challenges lie in data quality and fitness for (quantitative) clustering.

## Introduction

Although eHealth technologies are seen as an opportunity to support self-management and disease management for patients with chronic diseases, their actual use remains low [1]. As a result, the full potential of eHealth technologies is not being reached. A possible explanation for these lacking applications is that during the development process, insufficient attention is paid to the needs, wishes, and context of the prospective end users. To overcome such issues, user-centered design (UCD) principles [2] provide tools to keep the intended user in the heart of the eHealth development process. The UCD practice of creating personas is widely accepted to ensure the fit between a technology and the target group or end users throughout all phases of development [3]. Personas represent fictive members of the target group and consist of a description of these potential users. By engaging with the personas, developers and project team members develop an eye for the characteristics of their target group [4]. One could say personas are a way to continuously communicate “who we are doing this for” to the team. In addition, the eHealth development team can, for example, anticipate on these personas to tailor educational messages [5] or to support adherence among several types of eHealth users [6]. The approaches that are described for creating personas are using 1 source of data, ignoring the variety and variability in data needed to create groups of end users that have similar characteristics.

Several frameworks advocate the use of multiple methods for data collection during the eHealth development process [7], for example, through interviews, questionnaires, and focus groups. Thus, mixed types of data from several sources are used during eHealth development, while persona creation often relies on limited data sources. First, the target group’s health-related attributes form an important part of the personas in eHealth projects [8]: the risk of health complications, health-related activities that the prospective end users must undertake, the variation of symptoms in the target group, and tailoring options for medical treatment. These topics reflect factors that can be used to paint the “end user picture.” Thus, health-related factors are the major contributors to the construction of eHealth personas. However, as research and experience in eHealth development progresses and matures, it has become obvious that an eHealth user should be characterized by more than just health status, and zooming in on health-related factors only tells a part of the users’ story. Rather, a person who may be ill or has a chronic disease and aims to recover after surgery or disease or simply looks to preserve his/her health still has many more personal characteristics, likes, dislikes, or habits that are also relevant for understanding this person [9]. Therefore, second personas are created focusing on how a person wants, likes, or prefers to live life. LeRouge and colleagues [10] developed a conceptual model for identifying a broad range of user profiles and persona attributes from qualitative data. A related approach that considers characteristics beyond health factors is described by Vosbergen et al [5]. They have demonstrated how a variation in information needs can lead to personas (and consequently, technology design) that represent different ways in which people value and consume information. Similarly, there are many preferences, habits, and other variables beyond health/disease status and demographics that may be worthwhile to include in eHealth personas [11,12]. In these approaches, the personas result from a selection of relevant factors depending on more subjective experiences and tacit knowledge from experts. This can easily result in somewhat arbitrary decisions made on what to include in the persona. An approach that addresses this issue is proposed by Holden et al [13], using a quantitative cluster analysis on biopsychosocial survey data. In their approach, Holden et al [13] use qualitative data such as subjective eHealth literacy to describe the target group and distill personas that represent this group.

In addition to the use of health-related data and person-oriented data, we have noticed approaches in which server log data are used for identifying and describing user groups. Server log data are an automatic registration of, among others, the time, date, and activity that is carried out by the eHealth user within the system. An example is the identification of user groups based on activities within the eHealth system, resulting in personas characterized by activities that are most prominent within the clusters [14]. A more comprehensive approach is described in the study by Jones et al [15], in which activities within the system are expanded with information about the frequency, intensity, consistency, and demographics of the users. Using such data results in personas that include demographics of the users as well as users’ engagement with an eHealth system. When this method is applied for identifying groups of eHealth users with chronic conditions, this approach itself can be expanded with log data related to monitored health values.

Overall, we see that there are several frameworks describing the steps in a very structured or less structured manner through which eHealth technologies can be developed. These frameworks are similar in that we see several data collection methods during the phases that are iteratively walked through to come to a technology that fits with the end users. In this sense, applying a framework in eHealth development and persona creation alike benefits from applying a broad lens to the user, technology, and context to ensure a good fit. The Center for eHealth Research (CeHRes) roadmap [7] describes such an approach, where research and development are guided through various design phases. This approach calls for holistic and value-driven development, focusing not only on the functionality and goal of eHealth technology but also accounting for users’ motivations, abilities, circumstances, and context [7]. Personas fit well within this approach if we include relevant factors/characteristics for creating personas. However, the approaches for developing personas, as described above, only focus on 1 method for collecting data (eg, interviews, questionnaire data, log data), ignoring the variety of data collected during the UCD development processes. Therefore, we have studied how to develop a structured iterative approach for personas within the eHealth development process. Data from a previous study were used in which the phases of the CeHRes roadmap were completed, resulting in data that were collected through various methods (eg, interviews, questionnaires, log data).

## Methods

### Study Design

In this study, we have used a 3-step iterative approach to personas. In the first step, health-related data were used to develop the personas, using data from an electronic patient record (EPR). In the second step, these EPR data were enriched with person-related data that were gathered through interviews and questionnaires. In the third step, log data were added to the model to illustrate how personas can be further developed after log data are collected through a pilot study or after the eHealth technology is launched and actually used by the end user. From now on, we refer to this iterative approach to eHealth development as the Persona Approach Twente (PAT). During this illustration of PAT, the focus is also on (1) how the approaches as described by Holden et al [13] and LeRouge et al [10] can be combined enabling the use of several data collection methods (quantitative and qualitative) for describing user groups and (2) the use of semiautomated methods for grouping the end users so that the arbitrary approach applied in previous studies for developing person-related personas is replaced by a more systematic approach. Thereby, we have aimed to contribute to achieving the full potential of eHealth technologies for chronic diseases.

### Data Collection

Data collected in a previous study for the development of a telemonitoring application for people with heart failure were used, guided by the steps described in the CeHRes roadmap [7]. These data were gathered among 25 patients with mild to moderate chronic heart failure from the outpatient clinic of the Hospital Group Twente, Almelo and Hengelo, The Netherlands, of whom 13 were females (56%). Their mean age was 68 (SD 9) years, ranging between 46 and 82 years. Patients with a New York Heart Association (NYHA) functional classification 2 or 3 [16], with stable symptoms, and stable medication were included in this study. Persons admitted to the hospital within 1 month after data collection were excluded.

First, data from EPRs of the participants were used to collect health-related data such as NYHA classification and cerebrovascular accident or transient ischemic attack comorbidity. Second, quantitative data were collected through the 8-item eHealth Literacy Scale (eHEALS) questionnaire [17] to gain insight into the eHealth literacy status of the participants. Third, the 5-level 5-dimension Euro quality of life (EQ-5D-5L) questionnaire was used to gain insight into participants’ quality of life, consisting of mobility, self-care, usual activities, pain/discomfort, and anxiety/depression [18]. Moreover, qualitative data regarding experiences in living with heart failure, technology use and trust, and motivation were collected through interviews with the participants. Based on these data, the iMediSense telemonitoring system (2016, Thales) was developed in another study [19]. A pilot study was conducted that was clinically supervised by cardiologists and nurse practitioners. In this pilot, patients were instructed to conduct measurements at least once daily for 60 days: diastolic blood pressure, systolic blood pressure, heart rate, and body weight. Further, they filled out an heart failure symptoms questionnaire. When measurements exceeded predefined ranges, alarms were generated. Nurse practitioners were instructed to view the generated alarms and react accordingly. The log data regarding the appointed symptoms, the alarms during the pilot study, and usage log data were used for the secondary analysis in this study. In Table 1, the aforementioned data collection methods are coupled with the variables that were collected through these methods. The variables also display the number of participants for whom a variable is known. Owing to the secondary analysis of this data set, not all variables were present or assessed among all participants.

**Table 1:** Data collection methods used in this study coupled with the variables that were collected through these methods and the number of participants for whom a variable is known.

Method, collected data		Variables (n)	Clustering
**Electronic patient record**			
	Demographic	Gender (25), age (25)	Step 1 and 2 and 3
	Medical	Cerebrovascular incident or transient ischemic attack comorbidity (25), chronic obstructive pulmonary disease comorbidity (25), diabetes comorbidity (25), left ventricular ejection fraction (25), heart failure with reduced ejection fraction (left ventricular ejection fraction <40%) (25), ischemic heart disease (25), hypertension (25), atrial fibrillation (25), New York Heart Association 2 or New York Heart Association 3 (25), heart failure hospitalization (25), cardiac resynchronization therapy defibrillator (25), estimated glomerular filtration rate (25), implantable cardioverter defibrillator (25)	Step 1 and 2 and 3
**Interviews**			
	Technical	Smartphone ownership (23), personal computer ownership (22), tablet ownership (23), use of technology for entertainment (13), use of technology for social purposes (14), use of technology for gaining information (14)	Step 2 and 3
	Demographic	Education type (7), children (13), grandchildren (5), divorce (13), marital status (16), employment (22)	Step 2 and 3
	Health care specifics	Positive coping (25), negative coping (25), health-related goals (25), years ago diagnosed with heart failure (24)	Step 2 and 3
**eHealth Literacy Scale questionnaire**			
	Capacity for engaging in eHealth	eHealth literacy (22)	Step 2 and 3
**5-level 5-dimension Euro quality of life questionnaire**			
	Quality of life	Quality of life before using the telemonitoring technology (25)	Step 2 and 3
		Quality of life after using the telemonitoring technology (25)	Step 3
**Log data of the pilot study**			
	Usage log data	Start new measurement (25), send symptoms measurement (25), send physical measurements (25), open history of measurements (25), contact care provider (25), open profile page (25), open user manual (25)	Step 3
	Appointed symptoms	Restless, forgetful, and had a lacking concentration (25), a reduced effort level (25), a reduced appetite (25), a more than normal increase in fatigue (25), increased shortness of breath (25), cough or tickling cough (25), moisture in legs and abdominal distension (25), increased palpitations, fast paced heartbeat and chest pain (25)	Step 3
	Generated alarms	Alarm for systolic blood pressure (24), alarms for diastolic blood pressure (24), alarms for heart rate (24), alarms for weight (24)	Step 3

### Data Analysis

Before analyzing the data, the qualitative data collected through the semistructured interviews were coded by 2 independent coders (FS and JW) by using a combination of inductive and deductive coding [20]. First, the scheme of LeRouge et al [10] with codes related to technical, demographic, and health care specifics were used to code the interview data deductively. Subsequently, these codes were adapted and supplemented by means of inductive coding. After qualitative analysis, all resulting themes and variations were categorized into binary variables to enable cluster analysis. This means that if a theme consisted of several variations, multiple binary variables were created: 1 for every variation. For example, marital status was divided into 2 variables, namely, marriage (married or not married) and divorce (divorced or not divorced). Moreover, when a code was assigned to less than 5 quotes, then these were deleted from further analysis to reduce the influence of the missing values on the cluster results. Second, Shapiro-Wilk tests were performed to check whether variables were normally distributed [21]. We found that the variables age, capacity for engaging in eHealth, and estimated glomerular filtration rate were normally distributed (*P*>.05). The remaining variables were not normally distributed (*P*<.05) (Multimedia Appendix 1) and therefore log transformed before carrying out the cluster analyses.

Since data were both numerical and binary, distance matrices were created using Gower distances. Gower distances can handle these types of mixed data by using range-normalized Manhattan distances for quantitative data and Dice coefficient for nominal variables [22]. Subsequently, 3 cluster analyses were carried out using the Partitioning Around Medoids algorithm to develop personas related to 1 of the 3 steps in the PAT. A cluster analysis is a form of exploratory data analysis, where observations are divided into meaningful groups that share common characteristics. The Partitioning Around Medoids algorithm was chosen since it fits with Gower distances, and the medoids can be used as “representatives” for the translation of clusters to personas. Medoids refer to observations that fall within a cluster for which the average dissimilarity between it and all the other members of the cluster is minimal. By using these representatives, we limit the influence of extreme values among the participants.

The analyses were conducted on 3 distinct parts of the same data set: (1) health-related data, (2) qualitative and quantitative health- and person-related data, and (3) qualitative and quantitative health- and person-related data, enriched with log data collected during the pilot study. All analyses were carried out using RStudio [23] and the R Cluster package [24], and results were visualized using the Ggplot2 package [25]. To estimate the optimal number of clusters, the average silhouette method was used. After conducting the cluster analyses, the medoids of the resulting clusters were used to describe personas. Table 1 summarizes which variables were included in the analysis for every step (1-3).

### Ethics Approval

All participants gave permission for the use of these data and signed an informed consent form. Moreover, this study was ethically approved by the Behavioral, Management, and Social Sciences ethics committee (210111).

## Results

Three cluster analyses were carried out that align with data collected through the (1) EPR (2) data enriched with interview and questionnaire data, and (3) the aforementioned data enriched with log data.

### Clustering Health-Related Data

**Figure 1:**
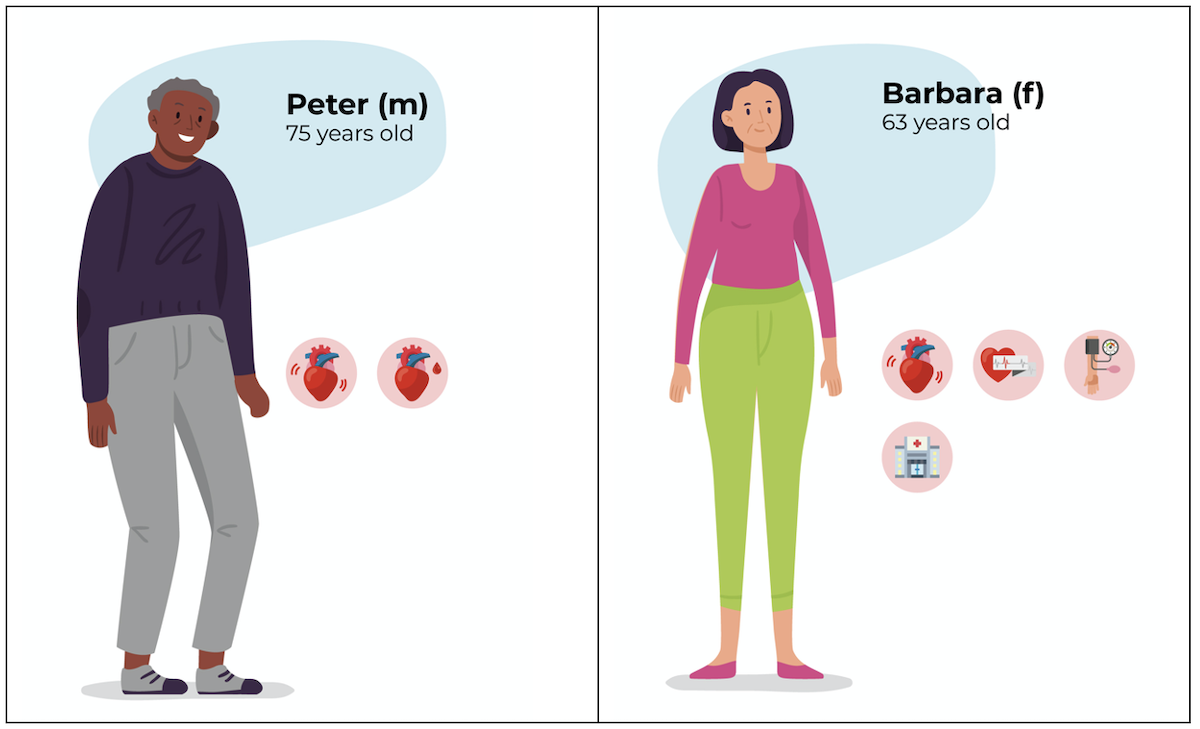
Personas developed in the first step on the basis of clustering electronic patient record data. Multimedia Appendix 3 shows the meaning of the symbols used in the persona descriptions. The red background indicates the medical characteristics. f: female; m: male.

Figure S1 of Multimedia Appendix 2 shows the average silhouette widths for the number of clusters ranging from 2 to 10. Based on this figure, it was decided that the optimal number of clusters was 2, yielding an average silhouette width of 0.17. In total, 25 persons were divided into 2 clusters. The first cluster has an average silhouette width of 0.12 and consists of 17 persons, which is 68% (17/25) of the total number of persons. The second cluster has an average silhouette width of 0.27 and consists of 8 persons, which is 32% (8/25) of the total number of persons. The medoids of these clusters were used to translate these clusters in personas. Two personas were created using the variable values of these medoids, and these can be found in Figure 1 (the meaning of the symbols used in the persona descriptions are given in Multimedia Appendix 3). The first persona is Peter (representing cluster 1), who has heart failure with reduced ejection fraction and an ischemic etiology. Second, the persona Barbara represents cluster 2, who has heart failure with reduced ejection fraction, hypertension, atrial fibrillation, and her estimated glomerular filtration rate was reduced (43 mL/min/1.73 m^2^). Barbara has had a prior hospitalization for heart failure.

### Clustering Health-Related Data Enriched With Person-Related Data

In the second step, we clustered the data set with health-related data, interview data, and the eHEALS questionnaire [17]. After the cluster analysis, an average silhouette plot was created yielding 2 clusters, and this can be found in Figure S2 of Multimedia Appendix 4. The corresponding average silhouette width for 2 clusters is 0.11.

**Figure 2:**
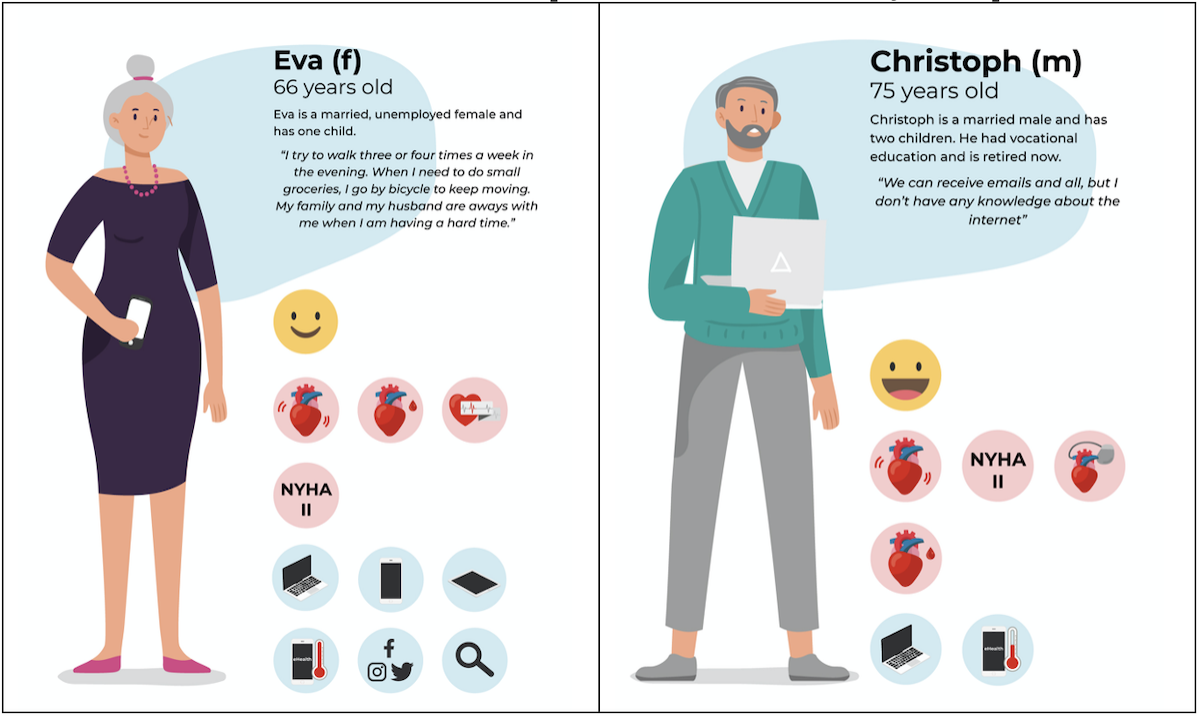
Personas developed in the second step on the basis of clustering electronic patient record data, data from the interviews, the eHealth Literacy Scale questionnaire, and the 5-dimension 5-level Euro quality of life questionnaire. Multimedia Appendix 3 shows the meaning of the symbols used in the persona descriptions. The red background indicates the medical characteristics, and the blue background indicates the technical characteristics. f: female; m: male.

Of the total of 25 persons, the first cluster consists of 10 persons (40%) with an average silhouette width of 0.08. The second cluster consists of 15 persons (60%) with an average silhouette width of 0.12. Persona descriptions were made based on the medoids within the 2 clusters, and these can be found in Figure 2. The first persona is Eva, who was diagnosed with heart failure with reduced ejection fraction of 33% and atrial fibrillation 2 years ago. Eva has a score of 10 on the EQ-5D-5L questionnaire on a scale from 5 to 25, indicating a good quality of life with slight problems or health issues. Eva mentioned 1 way of positive coping and 2 ways of negative coping. Eva owns a smartphone, computer, and a tablet. She uses this technology for social purposes (eg, social media) and for gaining information. Moreover, she has a mean score of 4 on the eHEALS questionnaire, indicating a moderately high capacity for engaging in eHealth. Correspondingly, Eva indicated that she has experience with eHealth technologies.

Christoph is a 75-year-old married male who had vocational education. He has 2 children and is currently unemployed. Christoph was diagnosed with heart failure with reduced ejection fraction 2 years ago. Besides, he has a left ventricular ejection fraction of 37% and an estimated glomerular filtration rate of 60 mL/min/1.73 m^2^. Christoph has ischemic heart disease. Christoph has an implantable cardiac resynchronization therapy defibrillator or an implantable cardioverter defibrillator to support his heart function. Christoph has a score of 5 on the EQ-5D-5L questionnaire, indicating a good quality of life. He mentioned 2 ways of negative coping with problems. Christoph owns a computer but no smartphone or tablet. Moreover, he has a score 3 on the eHEALS questionnaire, indicating a moderate capacity for engaging in eHealth. Moreover, Christoph indicated that he has no skills in working with eHealth technologies.

### Clustering Health- and Person-Related Data Combined With Log Data

**Figure 3:**
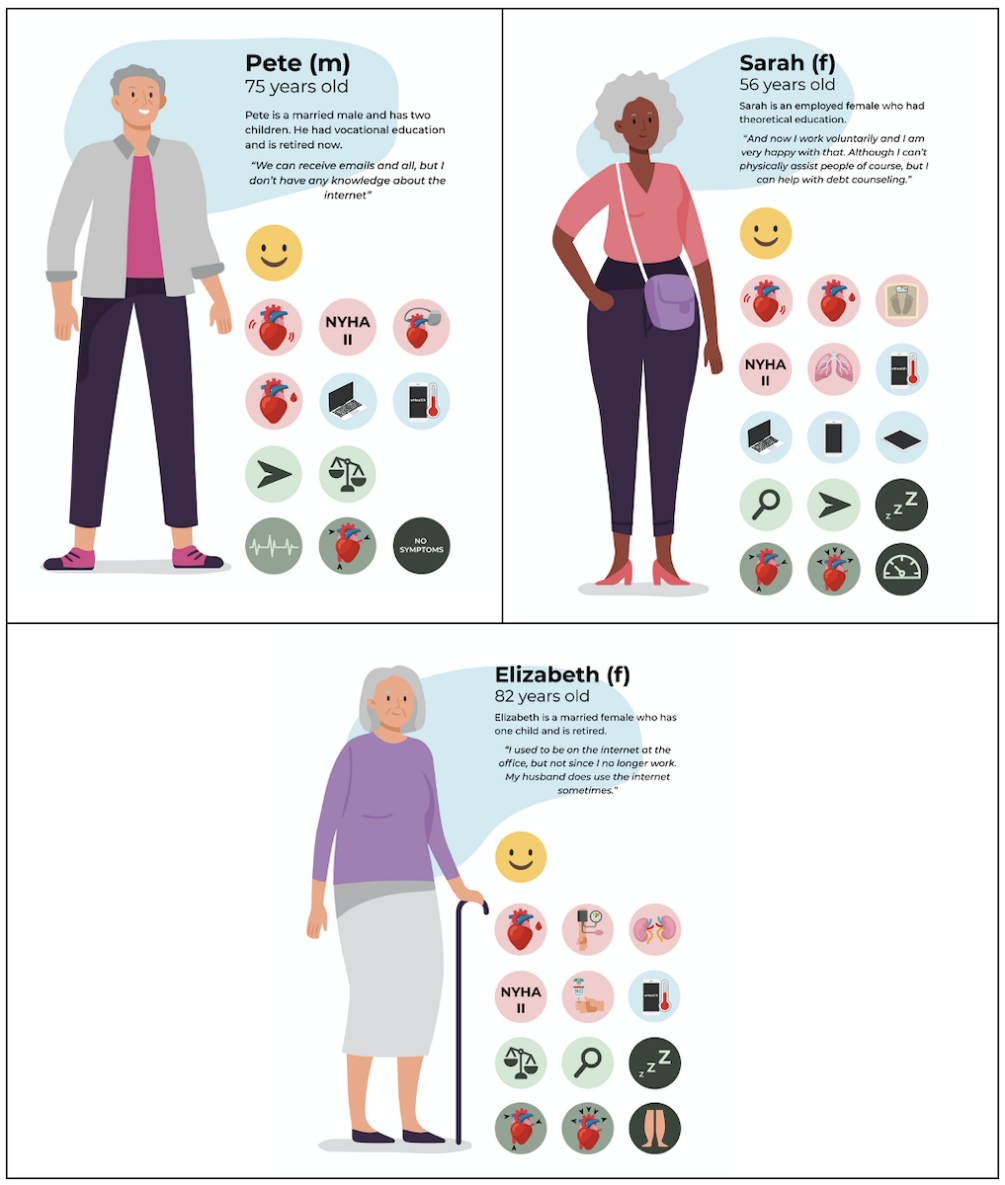
Personas developed in the third step on the basis of clustering electronic patient record data, data from the interviews, the eHealth Literacy Scale questionnaire, the 5-level 5-dimension Euro quality of life questionnaire, and log data. Multimedia Appendix 3 shows the meaning of the symbols used in the persona descriptions. The red background indicates the medical characteristics, the blue background indicates that the technical characteristics, and the green background indicates the log data from the pilot of iMediSense. f: female; m: male; NYHA: New York Heart Association.

In the third step, we enriched the health- and person-related data with usage log data that are typically collected after the design phase. After the cluster analysis, an average silhouette plot was created yielding 3 clusters. This average silhouette plot can be found in Figure S3 in Multimedia Appendix 5. The corresponding average silhouette width for 3 clusters is 0.08. Of the 25 persons, the first cluster consists of 15 persons (60%) with an average silhouette width of 0.09. The second cluster consists of 5 persons (20%) with an average silhouette width of 0.12. The third cluster consists of 5 persons (20%) with an average silhouette width of 0.04. Persona descriptions were made based on the medoids within the 3 clusters, and these can be found in Figure 3.

The first persona is Pete (representing cluster 1) who was diagnosed with heart failure with a reduced ejection fraction and an ischemic etiology 2 years ago. Pete did not mention any positive ways of coping and 2 ways of negative coping. Moreover, he has no smartphone or tablet, but he owns a computer. He had a score of 3 on the eHEALS questionnaire, indicating doubts in his skills to use information technology for health and mentioned that he has no skills in using eHealth technologies. During the pilot study, Pete indicated that he had no symptoms in the heart failure–symptoms questionnaire. Besides, alarms were mainly generated for heart rate (n=13) and diastolic blood pressure (n=10). During the pilot study, Pete showed a usage pattern in which only new measurements were started (n=77) and sent to the monitoring system (n=63). He visited his measurement history 1 time. Besides, he did not use other functionalities within iMediSense. His quality of life after using the monitoring system (EQ-5D-5L mean score of 5) did not change after using the monitoring technology.

Sarah represents cluster 2, and she was diagnosed with heart failure and reduced ejection fraction 1 year ago. Her estimated glomerular filtration rate was 88 mL/min/1.73 m^2^. Sarah has chronic obstructive pulmonary disease comorbidity and her goal is to maintain a stable weight. Sarah mentioned 1 way of coping positively and 3 ways of negative coping. Moreover, she owns a smartphone, tablet, and a computer. She finds her own skills in using of information technology for health reasonably high (eHEALS mean score of 4) and indicated that she has experience with eHealth technologies, but she does not see an added value. During the pilot study, Sarah indicated a mixed pattern of symptoms through the heart failure–symptoms questionnaire. She mentioned that she was restless, forgetful, and lacked concentration (n=4); she had reduced effort level (n=5), a reduced appetite (n=4), a more than normal increase in fatigue (n=7), increased shortness of breath (n=3), and cough or tickling cough (n=2). Her quality of life increased slightly (EQ-5D-5L mean score of 13) compared to her quality of life before using iMediSense (EQ-5D-5L mean score of 12). During the pilot study, alarms were mainly generated for heartbeat (n=29). Besides, alarms for diastolic blood pressure were generated 13 times, and the alarms for systolic blood pressure were generated 17 times. In iMediSense, Sarah started a new measurement 52 times, sent the symptoms measurement 36 times, and the physical measurement 37 times. Besides, she opened her measurement history 54 times and opened her profile page 37 times. Furthermore, she visited other functionalities a few times.

The third persona is Elizabeth (representing cluster 3) who was diagnosed with heart failure 2 years ago. She has hypertension comorbid with diabetes. Moreover, she has an estimated glomerular filtration rate of 47 mL/min/1.73 m^2^ and has been hospitalized before the current visit. Elizabeth has a score 3 on the eHEALS questionnaire, indicating doubts in her skills to use information technology for health. The main symptom that Elizabeth mentioned through the heart failure–symptoms questionnaire was moisture in legs and abdominal distension (n=37). During the pilot study, alarms were almost daily generated for systolic blood pressure (n=58) and diastolic blood pressure (n=43). In a much lower amount, alarms were generated for heart rate (n=5) and for weight (n=1). In iMediSense, Elizabeth shows a usage pattern in which she mainly started new measurements (n=165), sent the symptoms measurements (n=68), and looked into her measurement history (n=87). Her quality of life increased a little (EQ-5D-5L mean score of 8) compared to her quality of life before using iMediSense (EQ-5D-5L mean score of 7).

## Discussion

### Principal Findings

The practice of creating personas is widely accepted to ensure the fit between a technology and the target group or end users throughout all phases of development. Our demonstration of PAT shows that this approach can be used for developing personas through clustering mixed data in an iterative way to align with the process of eHealth development. This way, the richness of the persona increases as the development of an eHealth technology continues, while the use of a clustering algorithm partially ensures that these are objectively determined. PAT has the advantage that (1) the use of medoids makes the results easy to interpret, (2) mixed data can be used, and (3) personas can be iteratively developed. Below, we will elaborate on these advantages, and lastly, we will describe the disadvantages that we have encountered, along with a possible solution and direction for the future.

For the development of personas with heart failure by using PAT, we have used medoids as a method to find representatives for each group of users that have similar characteristics (clusters). These medoids have a minimum dissimilarity with other patients in the same cluster. Data from this representative patient (medoid) can be used for describing the persona. Holden et al [13] used comparative statistical tests between clusters to see on which variables these clusters differ. Subsequently, only the means of the variables that significantly differ are used to describe the personas [13]. PAT has several advantages compared to this approach. The first is that it is also suitable when the number of participants is low (which often occurs in the UCD process) since comparative statistical tests are also highly dependent on the number of participants. Second, PAT is less labor-intensive since it does not require to conduct comparative statistical tests. Third, it is easier to interpret, for example, a mean value of 0.5 on gender is difficult to interpret, which does not occur when medoids are used.

Besides the use of medoids, PAT allows including mixed data for persona development. In our demonstration of PAT on data collected in a project guided by the steps of the CeHRes roadmap, we were able to include data collected through questionnaires, interviews, EPR, and log data. This way, a more holistic understanding of the users can be reached. Moreover, including mixed data can be seen as an application of method triangulation [26]. For example, the NYHA classification of patients was extracted from EPRs, which is a description of the severity of the heart failure based on symptoms, and this classification ranges from I (no symptoms or limitations) to IV (severe limitations). However, the symptoms that a patient experiences and the way in which this limits the patient in his or her daily life might be understood in a more holistic way when adding data that are collected through another method.

Lastly, we saw how PAT aligned with the order in which data collection methods were deployed in the Twente Teach project. When applied during the development process, personas can be constantly updated based on newly collected data. This constantly updating of personas overlaps with the concept of Digital Twins [27]. The difference is that the current approach is focused on an up-to-date description of users on a group level, whereas Digital Twins are applied on an individual level. This ensures that the persona remains applicable and clear in the complex process of eHealth development. However, we do argue that the concept of “adaptive intelligence” should also be applied when PAT is used. This means that the personas are developed using an algorithm but that they become meaningful when domain knowledge is used for translating these personas into practical implications for targeting the users of the eHealth technology.

Although we found several advantages of PAT in this study, the results show that the quality of clusters decreases when qualitative data from the interviews are used in the cluster analysis (as expressed by the lower silhouette width). This, however, does not mean that the interview data are invaluable. Rather, it may imply that attention should be paid to what kinds of data are available or should be collected and how these are collected. Typically, health-related data are present for all patients included in a study, whereas the collection of more person-oriented characteristics of our patients or user groups is less standardized and defined. We argue that information about the person should be included, as health-related variables are measured more often and often in a more structured way, making them easier to use. The variables that stretch beyond health and tell us more about our user as a person, his/her background, circumstances, abilities, motivations, and values are at least as valuable to measure and use to create personas. However, this study shows that data quality is an issue when modeling the personas, and this occurs more often in less standardized variables. This applies to many of the information types described in LeRouge et al’s framework [10], which focuses on a broader context of eHealth user characteristics [10]. For example, technology use (technical specifics) or information-seeking attitudes (health care specifics) are potentially very relevant but are constructs that are rarely part of a standard and standardized medical assessment. To be able to use such possibly relevant variables, they should be measured in a more structured way.

Another possible remedy to this decreasing quality of clusters when adding qualitative data is to use domain knowledge for deciding which variables should be included in the cluster analysis or to summarize multiple variables into 1 variable (eg, use feature engineering or a factor analysis). However, since targeting eHealth users based on more than 1 variable is associated with a higher effectiveness of interventions [28], we state that a more systematic collection of person-oriented characteristics should be preferred. We argue that the steps below should be iteratively completed during the eHealth development process. These are also applicable in other contexts (eg, other target groups, when data are collected in a different order):

1. Collect data using a variety of methods and make sure that person-related variables are collected in a structured way.
2. Check whether variables are normally distributed and adjust analysis accordingly.
3. Carry out a cluster analysis to group participants into similar clusters.
4. Describe the clusters based on medoids and draw personas on the basis of the data that are known of these medoids.
5. Add qualitative data from these medoids to these personas to increase the richness of the persona descriptions.
6. Use domain knowledge to translate the personas into practical implications for the eHealth system to better target the eHealth to the users.

### Limitations

Owing to the explorative design of this study, the small sample size of 1 clinical center, and the homogeneous sample accordingly, it remains unclear to what extent results can be generalized across patients with heart failure and other situations and groups of people. However, the focus of this study was to show how PAT might be used to develop personas; therefore, generalization was not a condition for useful results. Nevertheless, the question remains to what extent cluster results can still be used within a development process when collecting a larger amount of data from the group of end users. Moreover, usage log data of iMediSense could not be used because there was too little variation in that data: adherence was high (almost 100%) and the ways in which users could navigate through the platform were limited. It would be relevant to explore to what extent clustering results are of predictive value for the ways in which users navigate through a system, when indeed adherence and navigation patterns vary. Further, application of remote coaching and education to promote self-management may alter the clustering and predictive value of navigation through the system, which warrants further research.

### Future Work

In future research, we will develop personas, including a larger number of participants, thereby allowing to test this combined approach on a larger sample. Moreover, intended use will be coupled with these personas, and usage log data will be used to see whether participants use it as intended. By continuing our research this way, we hope to learn how to attune technological features to our user. We hypothesize that technology personas can inspire developers to put the right persuasive features [29] in the designs and tailor them accordingly to different users. Moreover, in this study, we focused on how users can be better targeted using the PAT method. Specific methods for targeting eHealth are personalization, tailoring, and adapting eHealth. In future research, we aim to carry out a systematic review into how eHealth technologies are personalized. More specifically, we aim to investigate what information from the user is collected to personalize the eHealth technology accordingly. Because we will also map out the effectiveness of these different types of personalization, we can also make a recommendation for the variables that should be considered when developing personas.

## Appendix

Multimedia Appendix 1 Results of the Shapiro-Wilk tests.

Multimedia Appendix 2 Average silhouette plot for the cluster analysis on the health-related data.

Multimedia Appendix 3 Meaning of the symbols in the persona descriptions.

Multimedia Appendix 4 Average silhouette plot for the cluster analysis on the health- and person-related data.

Multimedia Appendix 5 Average silhouette plot for the cluster analysis on the health- and person-related data enriched with usage log data.

## Abbreviations

CeHRes: Center for eHealth Research
eHEALS: eHealth Literacy Scale
EPR: electronic patient record
EQ-5D-5L: 5-level 5-dimension Euro quality of life
NYHA: New York Heart Association
PAT: Persona Approach Twente
UCD: user-centered design
