# Supplementary material for: Personas for Better Targeted eHealth Technologies: User-Centered Design Approach
Source: JMIR Hum Factors. 2022 Mar 15;9(1):e24172. doi: 10.2196/24172 (PMC8965674; doi:10.2196/24172)
Supplement: Multimedia Appendix 1 [file humanfactors_v9i1e24172_app1.docx]

## Appendix 1. Results Shapiro-Wilk tests

|  | Shapiro-Wilk | | |
| --- | --- | --- | --- |
|  | W | df | *P* value |
| Age | .97 | 25 | .57 |
| Woke up often, pee a lot during the night, and less during the day | .454 | 25 | .00 |
| Stuffy at night, extra pillows, sleeping on a chair | .22 | 24 | .00 |
| Restless, forgetful and lacking concentration | .38 | 25 | .00 |
| Reduced effort level | .65 | 25 | .00 |
| Reduced appetite, full feeling | .43 | 25 | .00 |
| More than normal increase in fatigue | .56 | 25 | .00 |
| Moisture in legs, abdominal distension | .42 | 25 | .00 |
| Increased shortness of breath | .51 | 25 | .00 |
| Increased palpitations, fast-paced heartbeat, chest pain | .57 | 25 | .00 |
| Cough, tickling cough | .68 | 25 | .00 |
| Left ventricular ejection fraction (LVEF) in % | .84 | 25 | .00 |
| Number of years ago diagnosed with HF | .74 | 24 | .00 |
| Quality of life before using iMediSense | .84 | 24 | .00 |
| Number of times the participant mentioned a positive way of coping during the interview | .55 | 25 | .00 |
| Number of times the participant mentioned a negative way of coping during the interview | .75 | 25 | .00 |
| Capacity for engaging in eHealth | .94 | 22 | .22 |
| Diastolic blood pressure | .87 | 24 | .01 |
| Heartbeat | .81 | 24 | .00 |
| Systolic blood pressure | .86 | 24 | .00 |
| Weight | .30 | 24 | .00 |
| Quality of life after using iMediSense | .86 | 24 | .00 |
| new-measurement | .78 | 25 | .00 |
| confirm button questionnaire (new-measurement) | .77 | 25 | .00 |
| confirm button send measurements (new measurement) | .78 | 25 | .00 |
| my-history | .28 | 25 | .00 |
| day button (measurements) | .67 | 25 | .00 |
| week button (measurements) | .65 | 25 | .00 |
| month button (measurements) | .66 | 25 | .00 |
| year button (measurements) | .66 | 25 | .00 |
| blood pressure button (measurements) | .46 | 25 | .00 |
| heartrate (measurements) | .66 | 25 | .00 |
| weight (measurements) | .53 | 25 | .00 |
| contact button (home) | .71 | 25 | .00 |
| my-settings | .71 | 25 | .00 |
| choose language button (settings) | .55 | 25 | .00 |
| absence button (settings) | .46 | 25 | .00 |
| user-manual-button (home) | .58 | 25 | .00 |
| estimated Glomerular Filtration Rate | .93 | 25 | .11 |
