# Supplementary figures and images for: Personas for Better Targeted eHealth Technologies: User-Centered Design Approach
Source: JMIR Hum Factors. 2022 Mar 15;9(1):e24172. doi: 10.2196/24172 (PMC8965674; doi:10.2196/24172)

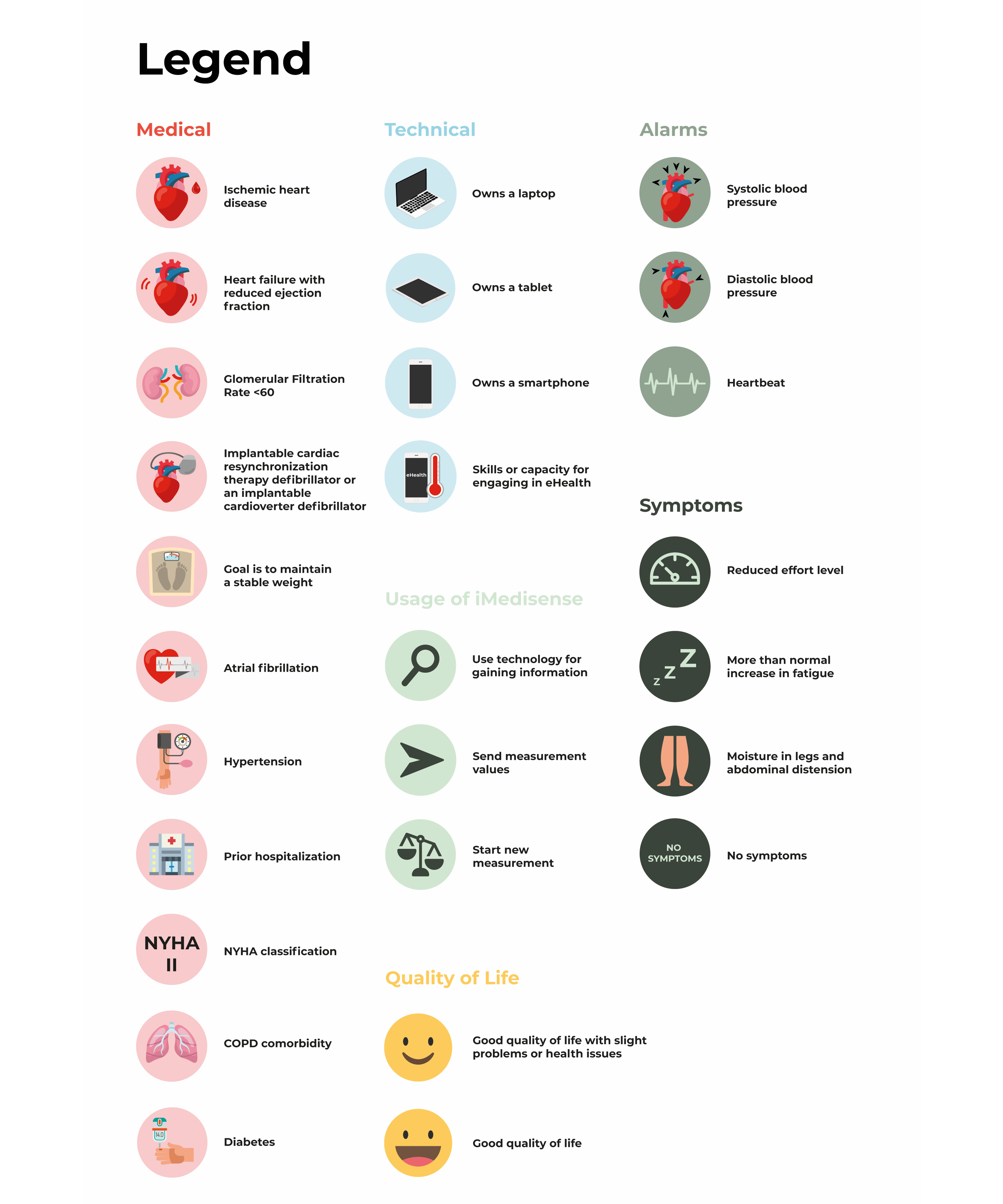

Supplement: Multimedia Appendix 3 [file humanfactors_v9i1e24172_app3.png]
