# Supplementary material for: Personas for Better Targeted eHealth Technologies: User-Centered Design Approach
Source: JMIR Hum Factors. 2022 Mar 15;9(1):e24172. doi: 10.2196/24172 (PMC8965674; doi:10.2196/24172)
Supplement: Multimedia Appendix 5 [file humanfactors_v9i1e24172_app5.docx]

## Appendix 5. Average silhouette plot for the cluster analysis on the health- and person-related data enriched with usage log data

Figure S3. Average silhouette plot for the cluster analysis on the health- and person-related data enriched with usage log data^a^.


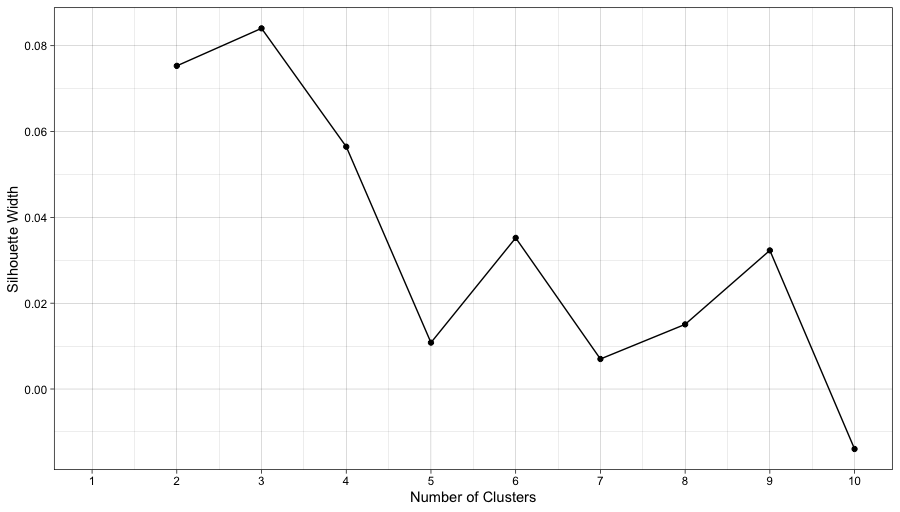


^a^ The x-axis shows the number of clusters ranging from 2 to 10, and the y-axis shows the corresponding average silhouette width, where a value of -1 indicates that the sample is close to its neighboring cluster, and a value of 1 indicates that the sample is far away from its neighboring cluster.
